# Direct or indirect: the crucial role of N-palmitate in hedgehog signaling

**DOI:** 10.1042/BST20260268

**Published:** 2026-05-15

**Authors:** Janna Puschmann, Kay Grobe

**Affiliations:** Department of Cellular and Molecular Glycobiology, Institute of Physiological Chemistry and Pathobiochemistry, University of Münster, Waldeyerstrasse 15, D-48149 Münster, Germany

**Keywords:** ADAM10, Dispatched, Drosophila melanogaster, hedgehog, palmitate, plasma lipoproteins

## Abstract

During development, cells use concentration gradients of soluble signaling molecules, called morphogens, to determine their position within tissues and adopt the correct identity. Hedgehog (Hh) morphogens are a key but also unusual example of gradient formation, because their diffusion away from their origin to direct growth and patterning is first prevented by their dual N- and C-terminal lipidation by palmitate and cholesterol. These lipids associate the Hhs with the plasma membrane of the cells that produce them. Later, however, both lipids participate in the spatiotemporal release of Hh by accessory proteins and maintain Hh biofunction *in vivo*, either directly or indirectly. The present study explores these two mechanistic possibilities, focusing on the central role of N-palmitate in Hh-regulated development in both vertebrates and invertebrates.

## Introduction

Despite the essential roles of the highly conserved Hh signaling pathway for embryonic development and adult physiology [1], some of the aspects that control Hedgehog (Hh) biofunction are still enigmatic. One of these aspects is that during their biosynthesis, all Hh family members (Hh in *Drosophila melanogaster* and Sonic Hh (Shh) in vertebrates) are covalently linked to a palmitate moiety at the protein’s N-terminus [2] and to a cholesterol moiety at the protein’s C-terminus [3]. As a consequence, both lipids firmly tether the signaling molecule to the plasma membrane (PM) of the cells that produce it to prevent its spontaneous transport away from the source. This is in notable contrast with most other signaling molecules that are either directly secreted or proteolytically removed from the surface of producing cells in a process called ectodomain shedding [4]. This paradoxical situation—that a paracrine signaling molecule is produced in a tightly PM-associated, insoluble form—raises the important question of how the Hhs overcome their firm PM association and spread to target cells at significant distance from the source. Equally important is the question of whether the lipids may play additional roles later in the Hh pathway, such as contributing to Hh signaling to its receptor Patched (Ptc in *Drosophila*, Ptch1 in vertebrates) on distant receiving cells. Both questions are interconnected, and therefore the answers to one question restrict the hypothetical possibilities to be taken into account to answer the second question. For example, if both lipids contribute to the Ptc/Ptch1 signaling process, Hhs must be extracted from the PM together with the lipids and will require dedicated soluble chaperones to transport them to the receptor. However, in the alternative scenario that PM-associated Hhs solubilize by specific removal of their lipid termini, any later role of these lipids in transport and signaling to Ptc/Ptch1 is excluded. Over the past two decades, structural, biochemical, and cell biological analyses have provided ample evidence supporting both modes. The current preference for one mode over the other depends on its perceived plausibility and the amount of direct or indirect experimental support it has. This review addresses those topics.

## Hh biosynthesis

The first posttranslational modification during Hh/Shh biosynthesis is an autocatalytic cleavage of the 45-kDa proprotein into a 19-kDa N-terminal signaling domain that will later become the bioactive ligand. This cleavage is performed by the C-terminal autoprocessing/cholesterol transferase domain that uses an intein-related mechanism to attach a cholesterol moiety to the C-terminus of the N-terminal signaling domain [3]. The second posttranslational Hh/Shh modification occurs at its N-terminus, resulting in the dually lipidated signaling proteins. In contrast with the quantitative C-terminal auto-cholesteroylation of Hhs, the N-palmitoylation step requires high enough expression of a separate enzyme called Hh acyltransferase (Hhat in vertebrates or skinny hedgehog (Ski) in invertebrates) [5,6]. This is followed by Hh export to the PM, where it localizes in sphingolipid- and cholesterol-rich areas called lipid rafts [7].

## The C-sterol: a mediator of Hh solubilization and controller of signaling range

*In vivo* experiments revealed that, in addition to their role in PM association, both Hh lipids play other important functional roles. The replacement of the C-cholesterol by a transmembrane (TM) domain or a glycosyl-phosphatidyl-inositol (GPI) anchor disrupts Hh removal from the PM and its transport to distant target cells [8,9]. This shows that C-cholesterol plays a facilitating role for Hh release from the PM of producing cells and for its subsequent transport, and suggests that an accessory protein removes and transports the C-sterol, but not other membrane anchors, from the PM of Hh-producing cells. This accessory protein is the 12-pass TM protein Dispatched (Disp) [9–12]. Disp carries a sterol sensing domain (SSD) [9] that shares structural homology with SSDs of hydroxymethylglutaryl-CoA reductase, Niemann-Pick-protein C1, SREBP cleavage-activating protein, and Ptc/Ptch1 or its orthologs in the worm [13–15], all of which sense or pump sterols. Consistent with the Disp SSD specifically releasing the Hh C-cholesterol from the PM, mutated recombinant proteins termed HhN in invertebrates or ShhN in vertebrates that lack the sterol are released in a Disp-independent manner and show a substantial increase of the signaling range [16–19]. Together, these *in vivo* findings show that C-cholesterol serves to retain the Hh ligand at the PM of producing cells, with the specific purpose to later use the dedicated release factor Disp to initiate spatio-temporally controlled Hh release and gradient formation in the extracellular space. In contrast, sterol is not essential for Ptc/Ptch1 receptor binding and activation. This is evident from the bioactivity of non-cholesteroylated HhN/ShhN and the *in vivo* TM- or GPI-linked Hh signaling to direct neighbors [8,9,20]. An important question arising from this SSD-dependent release mode is whether Disp also extracts the N-palmitate of Hh or Shh. While this is generally assumed to be the case, experimental evidence refutes this assumption because the expression of the transgenic ShhN protein, which is N-palmitoylated but lacks C-cholesterol, rescues many early Shh-related embryonic defects in Disp mutant mice [21]. This *in vivo* finding shows that Disp is not required to extract the N-palmitate of Hh/Shh from the PM, and the *in vitro* solubilization of ShhN is also Disp-independent [22,23].

## The N-palmitate: a determinant of Hh bioactivity

**Figure 1:**
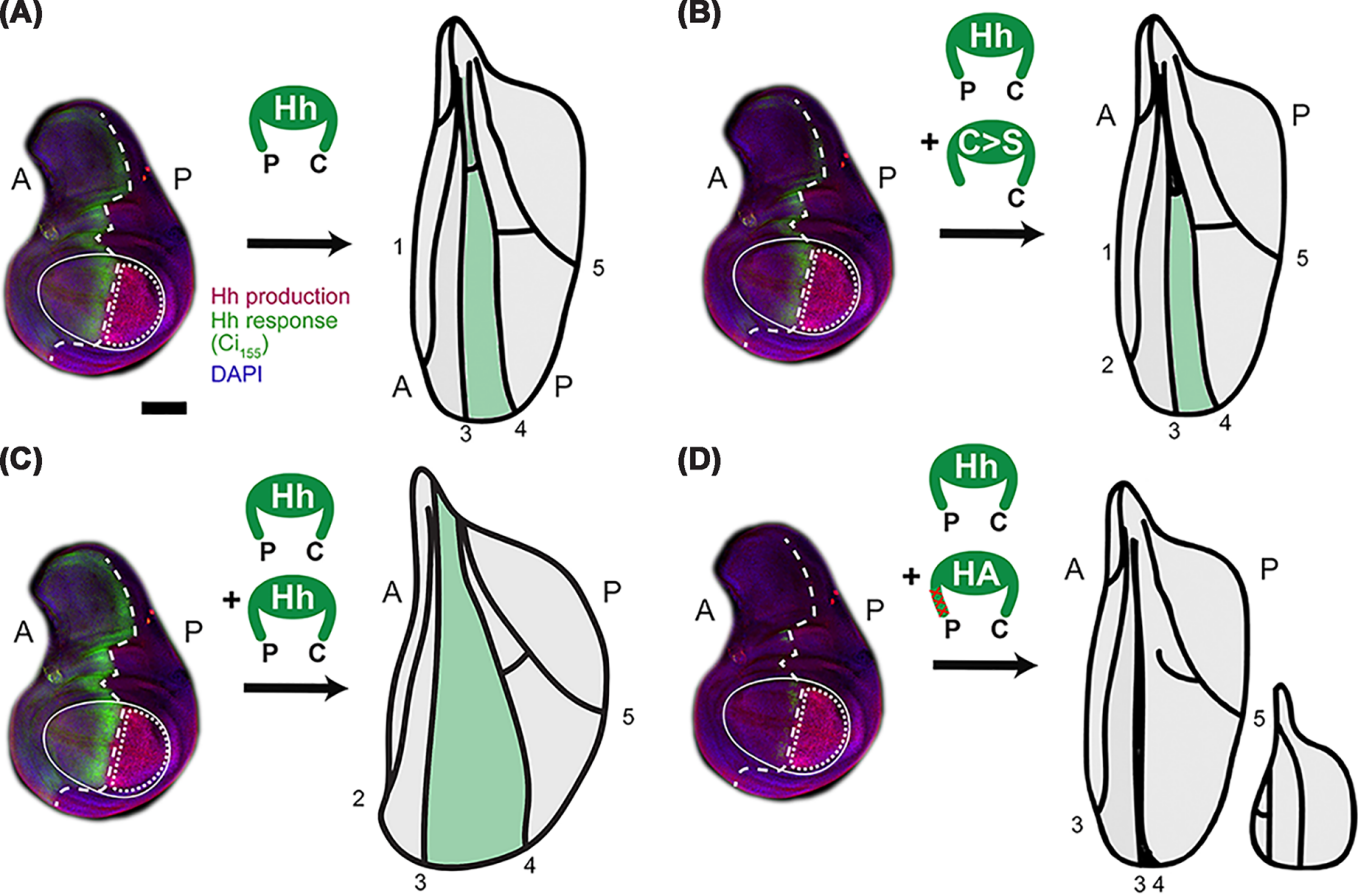
Hh-regulated patterning in the *Drosophila* wing disc depends on the palmitoylated N-peptide (**A**) The fly wing develops from the imaginal wing disc (left). The wing primordium, located at the center of the disc (solid circle), differentiates into the wing blade proper (shown in the cartoon on the right). This blade exhibits a distinctive pattern of five longitudinal veins (L1–L5). Hh is produced and released in the posterior (P) wing disc compartment (dotted area; Hh-producing cells are labeled red) and moves across the anterior/posterior boundary (dashed line) into the anterior (A) compartment. There, it signals to Ptc, leading to the accumulation of Ci155, which transcriptionally activates Hh target genes (the area of Ci155 expression is shown as a green anterior stripe). This process directly patterns the central L3–L4 region of the wing (artificially colored in green in the cartoon). (**B**) Coexpression of C>S Hh together with endogenous Hh in the posterior compartment reduces Ci155 accumulation and apposes the L3–L4 veins proximally in a dominant-negative manner [26,28,29]. (**C**) Conversely, when transgenic dual-lipidated Hh is co-expressed with endogenous Hh in the posterior wing disc compartment, it increases Ci155 and expands the L3–L4 intervein area. (**D**) Hh activity is not attributable to N-palmitate alone because inserting a protease-resistant HA tag downstream of the palmitoylated cysteine (shown red in the cartoon) and expressing dually lipidated HA-Hh in the posterior wing disc compartment abolishes Ci155 accumulation (left), possibly by stalling Disp export of the recombinant and endogenous proteins. This results in wings that lack most of the L3–L4 intervein area or even the entire anterior wing region (right) that both require Hh. These phenotypes copy complete Hh loss of function in the wing disc. Scale bar: 100 μm.

The functional role of the N-palmitate is, therefore, different from that of the sterol. Indeed, Hh and Shh variants made defective in N-palmitoylation during biosynthesis, either by Hhat/Ski removal or by the removal of the acceptor cysteine, resulting in artificial proteins called C>S Hh in *Drosophila* and C>S Shh in vertebrates, are released by Disp based on their complete cholesteroylation, but show diminished signaling *in vivo* [6,16,24,25]. Yet, C>S Shh and C>S Hh can also act as hypomorphic proteins in some contexts in the mouse [26] and in the fly [27]. Another striking property of palmitate deletion during biosynthesis is that artificial C>S Hh impairs the bioactivity of endogenous Hh in a dominant-negative manner when both proteins are expressed in the same *Drosophila* wing disc cells [23,28,29] (Figure 1A,B). In contrast, dual-lipidated Hh co-expression in the same cells enhances Hh signaling as expected (Figure 1C). In this experimental *in vivo* system, Hh gain-of-function or loss-of-function can conveniently be assessed by the size of the central space of the adult wing, because the size of this space correlates directly with Hh signaling activity during disc development. Therefore, an increased tissue area in adult wings reliably indicates increased Hh signaling during development, while a smaller central area relative to the wild-type wing indicates Hh loss of function (Figure 1A–C, represented by the green stripe at the anterior/posterior border). Together with the finding that the non-acylated proteins are efficiently secreted and reach their targets [6], the general conclusion from these experiments is that Hh palmitoylation must be a modification vital for Ptc binding and Ptc-controlled Hh signaling *in vivo*. This possibility is supported by structural analyses of the Ptch1 receptor together with dually lipidated Shh that was co-expressed or detergent-extracted from Shh-transfected cells [30,31]. These cryo-EM co-structures showed that Shh can bind Ptch1 via the globular protein domain together with the palmitate and cholesterol moieties, with the palmitate potentially contributing directly to Shh signaling. This latter finding in particular was considered as proof that the Hhs must be extracted from the PM together with their lipids, while ruling out mechanisms that would release the Hhs without the N-terminal palmitate.

## Not so fast: an alternative role for N-palmitate in restricting Hh release

A recent publication has challenged this consideration by showing that Shh release involves the cell-surface sheddase, a disintegrin and metalloproteinase 10 (ADAM10) [23]. In this release mode, the Disp protein first removes the cholesterol-modified Shh peptide from the PM and transfers it to a soluble chaperone. Then, Shh extraction is finalized by the proteolytic processing of the N-terminal peptide that holds the palmitate PM anchor [32]. Once these steps are finished, Disp is free to associate with the next Shh C-terminal cholesterol and extract it from the PM, followed by another step of N-processing and Shh solubilization. While such N-processed soluble morphogens would be expected to lack activity based on the aforementioned considerations, similar amounts of cholesteroylated Shh expressed in serum presence exhibit similar bioactivities *in vitro* regardless of the presence or absence of palmitate [2]. This long-standing observation has recently been reproduced independently in two different reporter cell lines [23,32], which reveals discrepancies regarding the functional importance of palmitate for Ptch1 activity regulation *in vivo* and *in vitro*. Importantly, ADAM10 processing of the palmitoylated N-peptide is not a cell culture artifact, because inhibition of the ADAM10 ortholog in *Drosophila* reduced Hh-dependent development of the compound eye to the same degree as in flies lacking Disp or Hh expression [32]. Like in the developing wing, the amount of eye tissue formed in response to Hh (here, the number of photoreceptors called ommatidiae, which can be easily counted) is a reliable readout for Hh activity during development. The same strong loss-of-function eye phenotype observed for ADAM10 ortholog deletion is seen when its N-terminal Hh cleavage site is blocked by a protease-resistant hemagglutinin tag (HA-Hh) [28,29,33]. Most notably, endogenous Hh bioactivity is completely blocked when HA-Hh is expressed in the *Drosophila* wing disc (Figure 1D) [28,29]. Along the same line, co-expressing ADAM10-resistant HA-Hh together with transgenic Hh—which, when expressed alone, results in strong gain-of-function phenotypes (Figure 1C)—did not reverse the striking dominant-negative phenotype caused by HA-Hh [28,29] (Figure 1D). The most parsimonious explanation for these results is that the N-terminal HA-tag located at the cleavage site blocks the final release step by the *Drosophila* ADAM10 ortholog. Consequently, the stalled transfer prevents Disp from completing the export cycle for recombinant or endogenous proteins, which progressively diminishes the level of Hh-accessible and functional Disp [23]. This means that, while these experimental results confirm the notion that N-palmitate contributes significantly to Hh and Shh bioactivity, palmitate does not necessarily signal directly to Ptc/Ptch1 both *in vivo* and *in vitro*. Instead, the described eye and wing phenotypes suggest that palmitate’s role as a membrane anchor is its only role: Its spatiotemporal proteolytic cleavage controls and completes Disp-mediated Hh/Shh transfer to a soluble acceptor.

Consistent with this idea, it is worth noting that the strong dominant-negative effect of HA-Hh expression can be reversed by the additional deletion of the palmitate PM anchor [28,29]. This additional mutation converts the extreme dominant-negative effect of HA-Hh on endogenous Hh shown in Figure 1D into the much milder wing phenotype as shown in Figure 1B, supporting that the unprocessed lipidated N-terminus anchors HA-Hh to the PM and stalls the Disp export machinery [28,29]. It also shows that the N-palmitate does not get passively pulled out of the PM by Disp, because this would not result in dominant negative phenotypes but rather in the Hh gain-of-function phenotype shown in Figure 1C. Another interesting aspect of ADAM10-mediated and N-palmitate-controlled Hh transfer mode is that, if C-cholesterol extraction by Disp occurs first and is followed by its transfer to a soluble chaperone, force is exerted on the N-peptide. This may expose the N-terminal peptide processing site, which is called the “proteolytic switch domain” in other proteins. This is a common theme in the mechano-regulation of ADAM10-mediated substrate cleavage: a pulling force is the key switch to activate shedding, as has been observed for Notch as well as for other ADAM10 substrates [34,35]. In the case of Hhs, the Disp protein may exert a pulling force during C-terminal peptide transfer to the soluble chaperone. This facilitates ADAM10 cleavage of the N-peptide and times the release.

Nevertheless, a central question regarding the reported *in vivo* phenotypes remains: How can the reduced bioactivity and mild dominant-negative activity of non-palmitoylated C>S Hh and C>S Shh variants be explained if they are not due to reduced binding and signaling to the Ptc/Ptch1 receptors?

## The importance of precisely timed Hh release

**Figure 2:**
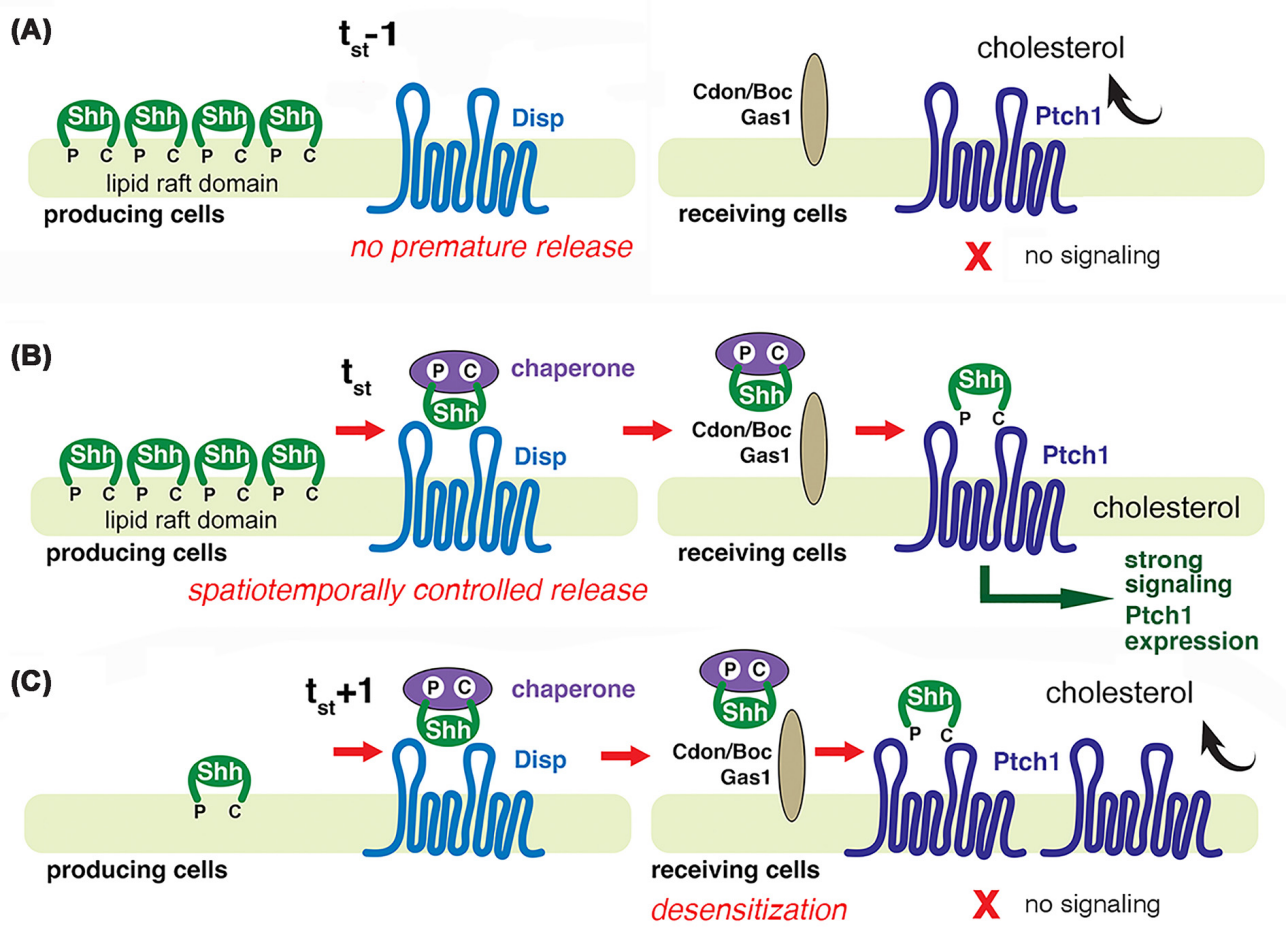
Shh solubilization and signaling (**A**) Before physiologically controlled Shh release occurs (t_st_ − 1), the Ptch1 receptor removes or redistributes cholesterol, a second messenger, from the PM. This inhibits downstream signaling and accumulation of the Ci155 ortholog Gli (as indicated by the red X). (**B**) Precise spatiotemporal control (at t_st_) ensures that target cells are exposed to Shh at relevant time points and for specific periods of time. Disp releases Shh, and accessory factors act as chaperones for the lipophilic adducts. On the target cell, Shh is transferred via coreceptors (Cdon, Boc, and Gas1) to Ptch1. Upon ligand binding, the cholesterol redistribution of Ptch1 is inhibited, the second messenger accumulates in the PM, and downstream components of the Shh signaling pathway are activated, including Gli. (**C**) The Ptch1 receptor becomes up-regulated in cells and tissues that receive the Shh signal. Ptch1 accumulation in these cells then sequesters later arriving (t_st_ + 1) Shh ligands and re-suppresses downstream Shh signaling by increasing the redistribution of the cholesterol second messenger. Therefore, after the initial spatiotemporal solubilization and signaling of Shh (t_st_), the pathway becomes less responsive to subsequent ligands (t_st_ + 1, red X) and is effectively shut down. t_st_: physiological spatio-temporal timing of Shh release and signaling. t_st_ + 1: Shh release from the same site at a later time. The Ptc rheostat function in flies is similar.

One possible answer is that, in vertebrates, the exposure of target cells to defined Shh concentrations and the timing of this exposure are crucial determinants of the response of these cells [36–42], and these two determinants depend on precisely controlled dynamics of morphogen release (Figure 2A,B). In the receiving target cell, solubilized ligand is then transferred via the coreceptors CAM-related/down-regulated by oncogenes (Cdon), brother of Cdon (Boc), and growth arrest-specific protein 1 (Gas1) to the receptor Ptch1 [43]. In the absence of the ligand at t_st_ − 1 (t_st_ stands for the time point of physiologically controlled Shh release (Figure 2A)), Ptch1 actively removes or redistributes PM cholesterol. Cholesterol in the PM otherwise acts as a second messenger to induce downstream signaling [13], so ligand-free Ptch1 keeps the Shh signaling pathway on the receiving cell silent. However, when exposed to the solubilized ligand, Shh-associated Ptch1 is blocked in its ability to redistribute cholesterol, thereby increasing its availability as a second messenger to induce signaling (Figure 2B). Importantly, the Ptch1 receptor acts as a rheostat, becoming up-regulated in cells and tissues that have received the Shh signal at t_st_ through a feedback loop, such as the developing vertebrate neural tube [44–47]. When Shh binds to Ptch1, it activates the pathway and, because Ptch1 itself is a target of Shh signaling, high levels of Ptch1 are induced in these same target cells (Figure 2C, right). Accumulating receptor then acts as a “sink” to sequester subsequently arriving Shh ligands at t_st_ + 1, thereby limiting their further spread and restricting gradient range. Furthermore, the accumulation of ligand-free Ptch1 resuppresses downstream Hh signaling by the active removal of the second messenger cholesterol, effectively rendering the target cell less responsive to later-arriving Shh ligands. This means that, in effect, target cells adapt temporally to the morphogens by integrating the concentration and duration of the signal to control differential gene expression [48]. This makes the precisely controlled dynamics of morphogen release and their amounts at t_st_ key determinants of cellular adaptation and, ultimately, the target cell response. Similar ligand-induced up-regulation of the Ptc receptor has also been observed in invertebrates, suggesting that precise spatio-temporal Hh release is equally relevant for unperturbed gradient formation and tissue patterning in the fruit fly [49–51].

## N-palmitate deletion during biosynthesis is unlike spatiotemporal delipidation during Hh/Shh release

**Figure 3:**
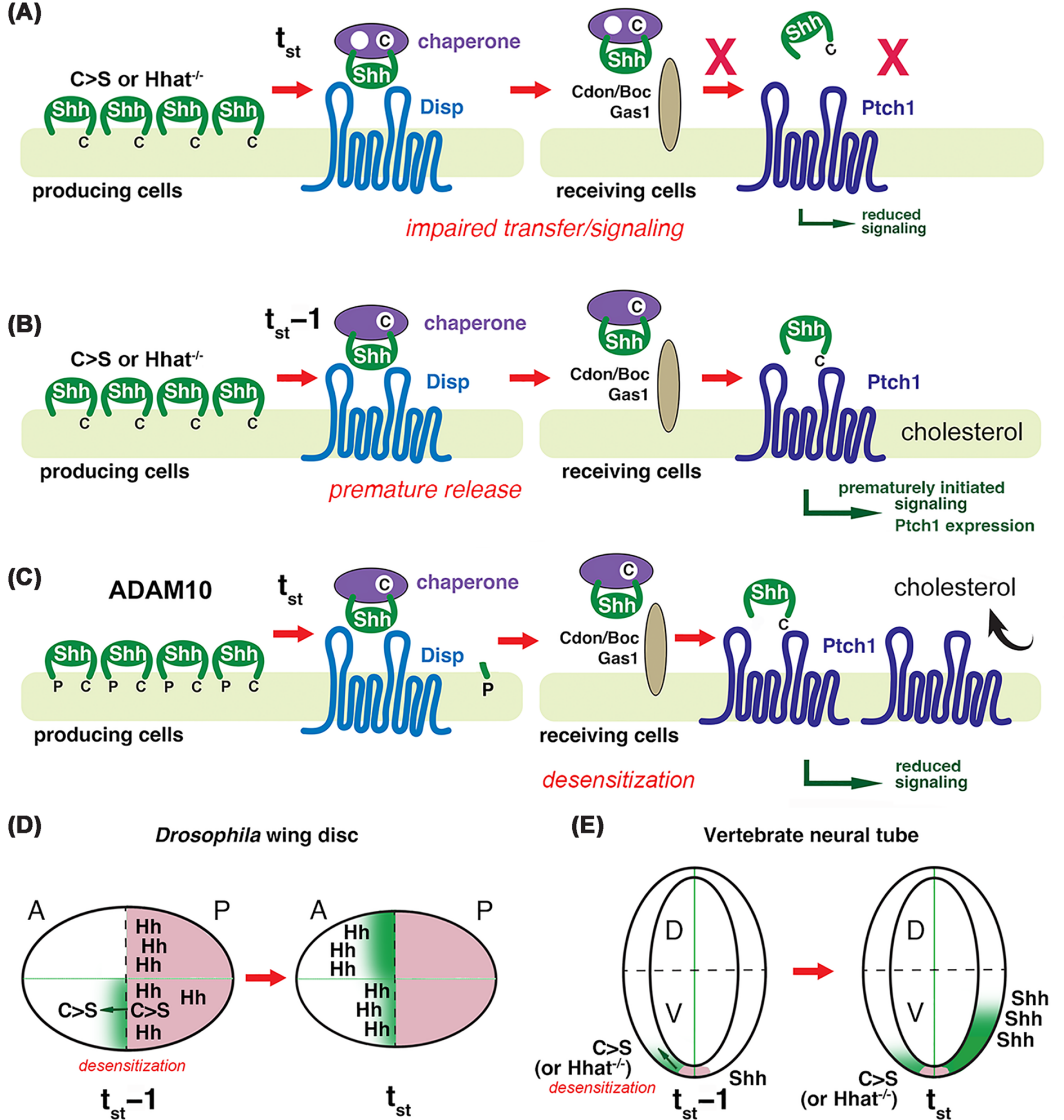
Altered solubilization and signaling of non-palmitoylated Shh variants (**A**) Variably reduced bioactivities and dominant-negative *in vivo* functions of artificially produced C>S Shh on endogenous dual-lipidated Shh biofunction, as currently explained. Compared with physiological Shh solubilization and signaling, the reduced bioactivities of non-palmitoylated C>S Shh are explained by reduced modulation of Ptch1 activity, assuming morphogen release at the correct time (t_st_). This may be due to impaired ligand handover from the soluble chaperone to co-receptors and/or from co-receptors to Ptch1, due to incomplete Ptch1 activity regulation, or both. (**B**) A possible alternative explanation for the variably reduced bioactivities and dominant-negative *in vivo* effects of non-palmitoylated C>S Shh. Unlike the dual-lipidated protein, C>S Shh is not safely stored in punctate PM release sites, but rather leaks prematurely from the PM (at t_st_ − 1) [23]. (**C**) This leakage up-regulates Ptch1 in receiving cells and tissues. This, in turn, renders receiving tissues less sensitive to the reduced amounts of Disp- and ADAM10-released physiological ligand to which they are exposed at the correct later time (t_st_). This reduces the signaling capacity of both the (leftover) artificial ligand and the endogenous Shh released at t_st_. Likewise, leakage of C>S Hh variants from the posterior wing disc compartment up-regulates Ptc in receiving anterior cells prematurely, which effectively limits the tissue’s response to the physiological ligand that arrives later. Possible consequences of C>S Hh/Shh leakage and tissue desensitization in the *Drosophila* wing disc (**D**) and the vertebrate neural tube (**E**). Premature induction of Ptc expression (labeled green) in the anterior compartment by C>S Hh released from the posterior wing disc compartment (bottom half) limits the spread and signaling response induced by later-arriving endogenous Hh. This could explain the apposition of the L3–L4 intervein area, which requires the highest dose of Hh signaling, as a consequence of C>S co-expression. Similarly, premature Ptch1 induction by C>S Shh leakage in the ventral neural tube (left half) may result in tissue desensitization. This, together with gradual depletion of the morphogen pool, could explain why C>S Shh signaling activity is variably reduced in most vertebrate tissues. A: anterior; P: posterior; D: dorsal; V: ventral. The field of Hh/Shh expression is labeled pink.

The variable loss-of-function and dominant-negative phenotypes observed in animals lacking Hhat/Ski or with a mutated ligand cysteine are currently explained by perturbed morphogen transfer or signaling to Ptc/Ptch1 [31,52] (Figure 3A). However, Hh/Shh palmitate function has historically been investigated through the selective mutagenesis of the N-terminal palmitate acceptor amino acid to generate C>S proteins, or the deletion of the Hh acyltransferase to produce non-acylated proteins. These proteins are secreted to the PM in a monolipidated form (Figure 3B), which renders them prone to immediate solubilization by Disp, unlike dual-lipidated Hhs, which accumulate at the PM [53] until their ADAM10-controlled release [23,32] (Figure 2A). The two settings that lead to monolipidated morphogens—one involving the immediate solubilization of artificially produced monolipidated C>S proteins and the other involving spatio-temporally controlled release of dual-lipidated Hh/Shh—result in different morphogen activities in space and time. Consequently, different patterning outcomes are produced. However, the differences between physiological delipidation and the experimental setup using C>S proteins, and their effect on the Hh gradient have not yet been considered an explanation for the highly variable *in vivo* phenotypes and their dominant-negative activities. Thus, the current hypothesis that the N-palmitate is essential for signaling to Ptc/Ptch1 is only one possibility for explaining its functional relevance. Another explanation is changed timing of developmental events, known as heterochrony, caused by the non-acylated proteins.

Indeed, the continuous “leakage” of bioactive C>S Hh and C>S Shh [2,23,32] has predictable effects on temporo-spatial Hh-regulated tissue patterning and proliferation *in vivo* (Figure 3B). The first is that the Ptc/Ptch1 rheostat becomes up-regulated in cells and tissues that receive the C>S signals prematurely (at t_st_ − 1), which then restricts the gradient range and signaling at t_st_ (Figure 3C) [49–51] as a consequence of the restored removal of the second messenger cholesterol [54]. Therefore, uncontrolled leakage of C>S Hh or C>S Shh renders receiving tissues unresponsive and numbs the ligand response at t_st_, while also limiting its spread by prematurely inducing Ptc or Ptch1 (at t_st_ − 1; note that palmitoylated Hhs are not released at this time) (Figure 2A). This mechanism of tissue “numbing” would effectively produce the same wide spectrum of developmental disorders that have been observed [36–41,48,55], yet with a different explanation than insufficient signaling to Ptc/Ptch1. Secondly, the premature continuous leakage of unpalmitoylated C>S ligands (at t_st_ − 1) depletes the PM-associated “reservoir” waiting to be released at the proper time (at t_st_). Both mechanisms can therefore explain the variably reduced activity of C>S Hh and C>S Shh *in vivo* [6,16,24,25], as well as their dominant-negative effects on co-expressed endogenous ligands [26,28,29] (Figure 3D,E). Importantly, this proposed scenario of target tissue desensitization via pre-exposure to a monolipidated morphogen that is constantly ‘leaking’ depends on whether it is bioactive. Although this is disputed, we would like to repeat that if Shh reporter cells are exposed to cholesterol-modified Shh that was produced in the same eukaryotic expression system in the presence of serum and normalized, their bioactivity is very similar regardless of whether palmitate is present or absent [2,23,32]. Furthermore, although nascent Hh/Shh associated with the PM is clearly dual-lipidated, direct and complete biochemical evidence of solubilized, dual-lipidated Hhs—the forms postulated to fully activate Ptc/Ptch1—has not yet been produced. In contrast, the conversion of dually lipidated, PM-associated Shh into the depalmitoylated product during ADAM10-controlled release has been demonstrated repeatedly [32,56,57].

## Can the inconsistencies between shedding and cryo-EM-derived structural data be resolved?

The final perceived drawback of proteolytic removal of the N-terminal palmitoylated peptide during Shh release is that cryo-EM structures showed that the Shh N-palmitate inserts into the cholesterol transport tunnel of Ptch1 to regulate the receptor [31,52]. As discussed before, this striking interaction is widely perceived as proof that the N-palmitate must not be removed during Shh release and transport, despite the aforementioned *in vitro* and *in vivo* results [2,23,28,29,32]. Nevertheless, there need not be a clash between ADAM10- and Disp-mediated depalmitoylated Shh release and the structural data. First, it is possible that more than one Shh transport and signaling mode exists, as shown by alternative cryo-EM structures that allow for Ptch1 activity regulation independently of the N-terminal palmitoylated peptide [14,58]. These structures are compatible with the bioactivity of non-palmitoylated ligands [2,23,32]. Second, the cryo-EM structure of Ptch1 and palmitoylated Shh [31,52] cannot disprove Shh depalmitoylation during its Disp-mediated release because the structure was artificially reconstituted using a dual-lipidated Shh ligand that was obtained by detergent extraction from Shh-transfected cells. Therefore, this ligand represents the PM-associated precursor protein in producing cells upstream of Disp and ADAM10 activity, which makes the question of how and in what lipidation state Hh/Shh ligands solubilize under physiological conditions even more central.

## Perspectives

- *Importance:* New findings show that, *in vitro*, deletion of the cell-surface-associated sheddase ADAM10 inhibits the solubilization of dually lipidated Shh from expressing cells to the same extent as deletion of the established Shh release factor, Disp. *In vivo*, suppression of endogenous ADAM10 activity in fruit flies inhibits Hh-dependent development of the compound eye to the same degree as functional deletion of Disp or the Hh morphogen itself. Biochemical characterization of the solubilized Shh protein revealed that it lacked its palmitoylated N-terminal peptide, though the C-terminal cholesterol remained present. These results support an updated Hh release and transport model. In this model, Disp only removes the cholesterol-modified C-terminal peptide of Hh and Shh to a soluble acceptor. The transfer is temporally controlled and completed by the ADAM10-mediated proteolytic removal of the N-terminal PM anchor.
- *Current thinking:* The current thinking is that Disp extracts both lipids from Shh or Hh and hands them over to a soluble chaperone. Then, the dually lipidated Hh/Shh ligand is handed over to the receptor, and the two lipids play functional roles in receptor signaling. This concept is supported by cryo-EM analysis of Ptch1 with a dual-lipidated Shh ligand that was PM-extracted from Shh-producing, transfected cells.
- *Future directions:* Although the question of whether Hhs are released in a monolipidated or dually lipidated form may seem minor, answering it is crucial for understanding how Hhs are released and transported. This, in turn, provides critical insight into the principles behind Hh gradient formation and its function as a morphogen rather than a simple signaling molecule. Over the past two decades, three potential modes of Hh/Shh release and transport from producing cells to Ptc/Ptch1 receptors on receiving cells have emerged: dual-lipidated Hh/Shh extraction and transport on soluble lipoproteins, Shh transport on soluble glycoproteins called Scube2, and Hh/Shh shedding by ADAM10, which is fundamentally different. Future research should focus on determining whether these seemingly competing mechanisms are all potentially required, alone or in combination, to generate lipidated or delipidated Hh ligands with different activities and signaling ranges from one PM-associated precursor.

## Open Access

Open access for this article was enabled by the participation of the University of Munster in an all-inclusive Read & Publish agreement with Portland Press and the Biochemical Society under an agreement with the TIB German Alliance.

## Abbreviations

ADAM10: a disintegrin and metalloproteinase 10
Boc: brother of Cdon
Cdon: CAM-related/down-regulated by oncogenes
Disp: Dispatched
GPI: glycosyl-phosphatidyl-inositol
Gas1: growth arrest-specific protein 1
Hh: Hedgehog
PM: plasma membrane
Shh: Sonic Hh
SSD: sterol sensing domain
TM: transmembrane
